# Analysis of influencing factors for progression from type 2 to type 1 in retinopathy of prematurity

**DOI:** 10.3389/fped.2026.1813325

**Published:** 2026-05-20

**Authors:** Shuyuan Zhang, Xingyue Chen, Jing Su, Wenfang Ye, Huan Ding, Kaiyan Zhang

**Affiliations:** 1 Department of Ophthalmology, Hainan Affiliated Hospital of Hainan Medical University (Hainan General Hospital), Haikou, Hainan, China; 2 Department of Neonatology, Hainan General Hospital (Hainan Affiliated Hospital of Hainan Medical University), Haikou, Hainan, China; 3 School of Public Health, Hainan Medical University, Haikou, Hainan, China; 4 Department of Ophthalmology, Hainan General Hospital (Hainan Affiliated Hospital of Hainan Medical University), Haikou, Hainan, China

**Keywords:** factors, preterm infants, retinopathy of prematurity, type 1 ROP, type 2 ROP

## Abstract

**Objective:**

To investigate factors associated with the progression of Retinopathy of Prematurity(ROP).

**Methods:**

A retrospective analysis was performed on 148 ROP infants. All cases were classified into type 1 and type 2 ROP according to the ETROP criteria, and those progressing from type 2 to type 1 were defined as progressive type (prog-type). Intergroup differences and relevant factors for prog-type were analyzed.

**Results:**

Among the 148 infants with ROP, 31 (20.9%) were type 1 ROP, 91 (61.5%) were type 2 ROP, and 26 (17.6%) were prog-type. Univariate analyses revealed significant differences among the three groups in gestational age (GA), birth weight(BW), delivery mode, 5-min Apgar score, hood/nasal cannula oxygen therapy(HH/MNCO) duration, non-invasive ventilation(NIV) duration, and presence of bronchopulmonary dysplasia(BPD) (all *P* < 0.05). The prog-type group had lower GA and BW, longer NIV duration and higher BPD rate. Significant differences in 5-min Apgar score and BPD existed between prog-type and type 2 (*P* < 0.05). HH/MNCO duration differed significantly between type 1 vs. type 2 and type 1 vs. prog-type (*P* < 0.05). Prog-type was most frequent in infants with GA of 27–32 weeks. In cesarean section (C-section) infants, the proportion of prog-type (12.8%) was significantly lower than that of type 1 (21.4%), while type 2 (65.8%) was significantly higher than type 1 (both *P* < 0.05). Type 2 was more common in the GA ≥ 33 weeks subgroup (*P* < 0.05). Multinomial Logistic regression identified GA (*OR* = 0.592, *95% CI* = 0.456–0.768, *P* < 0.001) and HH/MNCO duration (*OR* = 0.960, *95% CI* = 0.925–0.996, *P* *=* 0.029) as independently associated with prog-type, and HH/MNCO duration (*OR* = 0.954, *95% CI* = 0.918–0.992, *P* = 0.016) as independently associated with type 1 ROP (all *P* < 0.05).

**Conclusion:**

Different ROP groups exhibit distinct clinical characteristics. Lower GA, low BW, prolonged NIV and BPD were correlated with prog-type. Among C-section infants, prog-type was less common than type 1, whereas type 2 was more common than type 1. After multinomial adjustment, GA and HH/MNCO duration remained independently associated with prog-type, which may help clinical risk assessment and individualized intervention.

## 1 Introduction

Retinopathy of Prematurity (ROP) remains a leading cause of blindness worldwide among premature or low-birth-weight infants. Early detection and timely treatment can effectively prevent vision loss due to ROP (1). Risk factors for ROP have been well established through multiple evidence-based studies. Epidemiological data indicate that gestational age (GA) < 32 weeks, birth weight (BW) < 1,500 g, perinatal complications such as neonatal respiration distress syndrome (NRDS), bronchopulmonary dysplasia (BPD), and periventricular-intraventricular hemorrhage (PIVH), along with clinical interventions including oxygen therapy and blood transfusion, are all significantly associated with an increased risk of ROP (2–9). The Early Treatment for Retinopathy of Prematurity (ETROP) study established that management strategies differ according to ROP groups: infants with type 1 require timely treatment, whereas those with type 2 can be managed initially with close observation (10). In clinical ROP screening, we typically encounter three scenarios: some infants are diagnosed with type 2 at initial screening, some present with type 1 at the first visit, and others, initially classified as type 2, progress to type 1 during follow-up. Identifying the factors that drive progression form type 2 to type 1 may therefore be crucial for the timely prevention and management of ROP in high-risk infants. To address this, we retrospectively analyzed the clinical data of 148 infants diagnosed with ROP at our hospital between January 2014 and February 2025, aiming to investigate in depth the factors associated with progression from type 2 to type 1 ROP.

## 2 Methods

### 2.1 Subjects

Between January 2014 and February 2025, a total of 7,574 preterm infants (< 37 weeks) were admitted to the Neonatal Department of Hainan General Hospital. In accordance with China's national health care policy for children and following the guidelines for neonatal ocular disease screening (11), fundus examinations were performed on preterm infants with a GA of < 37 weeks at least once.

Ultimately, 188 infants were diagnosed with ROP.

The exclusion criteria included: death within 4 weeks after birth, lack of follow-up re-evaluation, and incomplete clinical data. After applying these criteria, 40 infants were excluded for the following reasons: 20 were born at other hospitals with unavailable postnatal diagnosis and treatment records; 6 underwent general anesthesia surgery due to systemic diseases; 13 were withdrawn from the study prematurely at parental request or due to incomplete follow-up; and 1 died during hospitalization. Consequently, 148 infants with ROP were enrolled in the final analysis. Screening timing and intervals were based on the 2014 Chinese Guidelines for ROP Screening (12).

Missing data were excluded from the final analysis, and no data imputation was performed.

### 2.2 Examination methods

To date, indirect ophthalmoscopy remains the gold standard and the most widely used screening modality for ROP (13). Prior to examination, mydriasis was induced using compound tropicamide eye drops. After adequate pupil dilation, topical anesthesia was administered with proparacaine hydrochloride eye drops, and the eyelid was retracted using a pediatric eyelid speculum. A comprehensive fundus examination was then performed by the same experienced retinal specialist using binocular indirect ophthalmoscopy with a 20-diopter lens. Diagnosis was made according to the ETROP criteria (10). The initial screening was initiated at 4–6 weeks after birth or at a corrected GA of 31–32 weeks, follow-up screening is performed every 2 weeks. If type 2 ROP is diagnosed, the screening interval is shortened to once weekly. Immediate treatment is required within 72 h once type 1 ROP is diagnosed (12).

### 2.3 Relevant definitions

In this study, the 148 infants with ROP were divided into three groups: type 1 ROP and type 2 ROP as defined by the ETROP study (10), and progressive type(prog-type), defined as infants who progressed from type 2 to type 1 during follow-up (e.g., zone II lesions developing plus disease, or zone I lesions progressing to stage 3 ROP. Aggressive ROP, characterized by rapidly progressive and ill-defined lesions, was classified as type 1 ROP in this study.

### 2.4 Data collection

Data were collected on the following 27 variables: gender, GA, BW, delivery mode, Apgar score, transfusion history, type and duration of oxygen therapy, intrauterine distress, premature rupture of membranes, placental abnormalities, umbilical cord abnormalities. Perinatal complications include NRDS, neonatal anemia, coagulopathy, hemolytic jaundice, neonatal pneumonia, cardiovascular disease and gastrointestinal disease. Maternal antenatal factors contained maternal age, hypertension, eclampsia, gestational hypertension and gestational diabetes mellitus.

Oxygen therapy modalities were classified strictly in accordance with the Chinese guidelines for the Care of Preterm Infants (2005) into three categories: head hood/modified nasal cannula oxygenation (HH/MNCO), noninvasive ventilation (NIV), and invasive mechanical ventilation (IMV). The duration (in days) for each type of oxygen therapy was recorded separately, with specific recording criteria as follows: for type 1 ROP, the cumulative duration of each oxygen therapy modality was recorded from birth to the date of the first diagnosis of type 1 ROP; for type 2 ROP, the cumulative duration of each oxygen therapy modality was recorded throughout their entire hospitalization; for prog-type, the cumulative applicable duration was recorded up to the time when timely treatment was identified.

### 2.5 Statistical analysis

Statistical analysis was performed using SPSS version 27.0 (IBM Corp., Armonk, USA). Continuous data were first tested for normality. For data that followed a normal distribution with homogeneous variance, one-way analysis of variance (ANOVA) was used, followed by Bonferroni correction for pairwise comparisons. For data that not following a normal distribution, the Kruskal–Wallis test was applied, also with Bonferroni correction for pairwise comparisons. Categorical data were expressed as frequencies (percentages) and compared among groups using the chi-square test. Pairwise comparisons among the three groups were further conducted with Bonferroni correction, and the adjusted significance threshold was set at *α* = 0.05/3 = 0.017. All statistical tests were two-tailed, with the overall significance level was set at *α* = 0.05.

## 3 Results

### 3.1 Comparison of clinical baseline data among the three groups

Of the 148 infants with ROP, 31(20.9%) were classified as type 1 ROP, 91(61.5%) as type 2 ROP, and 26(17.6%) as prog-type. Median GA was 31(29, 32) weeks for type 1 ROP, 31(29, 32) weeks for type 2 ROP, and 28(27, 31) weeks for prog-type, with a significant overall difference (*H* = 13.003, *P* = 0.012). Bonferroni-adjusted pairwise comparisons showed that prog-type differed significantly from both type 2 ROP (adjusted *P* = 0.002) and type 1 ROP (adjusted *P* = 0.006), while no difference was observed between type 1 and type 2. When examining GA subgroups (Tables 1, 2), within the type 2 group, the proportion of infants with GA < 27 weeks (2.2%, 2/91) was significantly lower than those with GA 27–32 weeks (80.2%, 73/91) and GA ≥ 33 weeks (17.6%,16/91) (both *P* < 0.05), with no difference between the latter two. within the type 1 and prog-type groups, no significant differences were found among the < 27 weeks, 27–32 weeks, and ≥ 33 weeks subgroups (all *P* > 0.05). Across GA subgroups, In the 27–32 weeks subgroup, type 1 ROP (21.2%, 25/118) was significantly more common than prog-type (16.9%, 20/118) (*P* < 0.05). In the ≥ 33 weeks subgroup, type 1 ROP (19.0%, 4/21) was significantly less common than both type 2 (76.2%, 16/21) and prog-type (4.8%, 1/21) (both *P* < 0.05), while no difference was found between type 2 and prog-type. No significant differences in ROP groups distribution were observed in the < 27 weeks subgroup (all *P* > 0.05).

**Table 1:** Comparison of clinical baseline data among three groups.

Variables		type 1 ROP (*n* = 31)	type 2 ROP (*n* = 91)	prog-type (*n* = 26)	Statistics	*P*
Gender	M	15 (48.6%)	56 (61.5%)	17 (65.4%)	*χ*^2^ = 2.118	0.347
	F	16 (51.6%)	35 (38.5%)	9 (34.6%)		
GA		31 (29,32)^a^	31 (29,32)^a^	28 (27,31)^b^	*H* = 13.003	**0**.**002**
GA subgroups					-	**0**.**020**
	< 27 w	2 (6.5%)^ab^	2 (2.2%)^b^	5 (19.2%)^a^		
	27–32 w	25 (80.6%)^a^	73 (80.2%)^a^	20 (76.9%)^a^		
	^3^ 33 w	4 (12.9%)^a^	16 (17.6%) ^a^	1 (3.8%)^a^		
BW		1,380 (1,130, 1,900)^a^	1,360 (1,050, 1,610)^a^	1,090 (915, 1,310)^b^	*F* = 4.478	**0**.**013**
Delivery Mode					χ^2^ = 8.912	**0**.**012**
	C-section	25 (80.6%)^ab^	77 (84.6%)^b^	15 (57.7%)^a^		
	vaginal	6 (19.4%)^ab^	14 (15.4%)^b^	11 (42.3%)^a^		
Apgar score						
	1 min	8 (6, 8)	8 (6, 9)	6 (4, 8)	*H* = 6.004	0.050
	5 min	8 (8, 9)^a^	8 (8, 9)^a^	8 (7, 8)^b^	*H* = 8.403	**0**.**015**
	10 min	8 (8, 9)	8 (8, 9)	8 (8, 9)	*H* = 5.438	0.066
Blood transfusion ^+^		21 (67.7%)	63 (69.2%)	22 (84.6%)	*H* = 2.645	0.266
Tot. O_2_ time, day						
	HH/MNCO	8 (3, 17)^a^	19 (5, 31)^a^	15 (8, 34)^a^	*H* = 6.197	**0**.**045**
	NIV	5 (0, 15)^a^	5 (0, 14)^a^	18 (8, 33)^b^	*H* = 12.887	**0**.**002**
	IMV	0 (0, 1)	0 (0, 1)	0.5 (0, 6)	*H* = 4.221	0.121
Intrauterine distress ^+^		3 (9.7%)	25 (27.5%)	6 (23.1%)	χ^2^ = 4.138	0.126
Premature Rupture of Membranes ^+^		7 (22.6%)	22 (24.2%)	2 (7.7%)	χ^2^ = 3.381	0.184
Placental Abnormalities ^+^		6 (19.4%)	16 (17.6%)	4 (15.4%)	χ^2^ = 0.154	0.926
Umbilical Cord Abnormalities ^+^		9 (29.0%)	17 (18.7%)	5 (19.2%)	χ^2^ = 1.552	0.460
Perinatal complications						
NRDS ^+^		16 (51.6%)	46 (50.5%)	20 (76.9%)	χ^2^ = 5.921	0.052
Neonatal Asphyxia ^+^		10 (32.3%)	38 (41.8%)	12 (46.2%)	χ^2^ = 1.278	0.528
BPD ^+^		4 (12.9%)^a^	21 (23.1%)^a^	13 (50.0%)^b^	χ^2^ = 10.410	**0**.**005**
PIVH^+^		5 (16.1%)	17 (18.7%)	4 (15.4%)	χ^2^ = 0.208	0.901
Sepsis ^+^		5 (16.1%)	15 (16.5%)	4 (15.4%)	χ^2^ = 0.018	0.991
Neonatal Anemia ^+^		12 (38.7%)	41 (45.1%)	10 (38.5%)	χ^2^ = 0.598	0.741
Coagulopathy ^+^		2 (6.5%)	8 (8.8%)	3 (1.5%)	χ^2^ = 0.563	0.841
Hemolytic Jaundice ^+^		1 (3.2%)	3 (3.3%)	0 (0.0%)	χ^2^ = 0.652	1.000
Neonatal Pneumonia ^+^		13 (41.9%)	48 (52.7%)	15 (57.7%)	χ^2^ = 1.590	0.452
Cardiovascular Disease ^+^		21 (67.7%)	61 (67.0%)	21 (80.8%)	χ^2^ = 1.867	0.393
Gastrointestinal Disease ^+^		6 (19.4%)	28 (30.8%)	12 (46.2%)	χ^2^ = 4.752	0.093
Maternal antenatal factors						
Age		30 (26, 32)	32 (28, 34)	30 (26.37)	*F* = 3.523	0.172
Hypertension ^+^		2 (6.5%)	10 (11.0%)	2 (7.7%)	χ^2^ = 0.469	0.855
Eclampsia ^+^		9 (29.0%)	28 (30.8%)	4 (15.4%)	χ^2^ = 2.424	0.298
Gestational Hypertension ^+^		3 (9.7%)	14 (15.4%)	2 (7.7%)	χ^2^ = 1.096	0.607
Gestational Diabetes Mellitus ^+^		2(6.5%)	10(11.0%)	6(23.1%)	χ^2^ = 3.585	0.175

+ Values are presented as n(%) of patients with the indicated condition. Continuous variables are presented as median and compared using the Kruskal–Wallis H test. Categorical variables are presented as n(%) and compared using the chi-square test or Fisher's exact test. After Bonferroni multiple comparison correction, the adjusted significance threshold was *α* = 0.05/3 = 0.017.

a,b The same superscript letter indicates no statistically significant difference between groups after Bonferroni correction (*P* > 0.017); different superscript letters indicate a statistically significant difference (*P* < 0.017). Bolded *p*-values indicate statistical significance.

**Table 2:** Pairwise comparisons among groups based on the Bonferroni method.

	GA			Delivery Mode		BPD	
	<27w	27–32w	≥33w	C-section	vaginal	Y	N
type 1	2^a^	25^a^	4^a^	25^a^	6^a^	4^a^	27^a^
type 2	2^a^	73^ab^	16^b^	77^b^	14^a^	21^a^	70^a^
prog-type	5^a^	20^b^	1^b^	15^b^	11^a^	13^b^	13^a^
Statistics	10.580			8.912	10.410		
*P*	**0.020**			**0.012**	**0.004**		

After Bonferroni multiple comparison correction, the adjusted significance threshold was *α* = 0.05/3 = 0.017.

^a,b^ The same superscript letter indicates no statistically significant difference between groups after Bonferroni correction (*P* > 0.017); different superscript letters indicate a statistically significant difference (*P* < 0.017). Bolded *p*-values indicate statistical significance.

Median BW was 1,380(1,130, 1,900) g for type 1 ROP, 1,360(1,050, 1,610) g for type 2 ROP, and 1,090(915, 1,310) g for prog-type (*F* = 4.478, *P* = 0.013). Bonferroni-adjusted comparisons showed a significant difference between prog-type and type 1 (adjusted *P* = 0.017, *95% CI* = 44.27–610.10), while no significant differences were found between the other groups (Table 1).

For delivery mode, the cesarean section (C-section) rates were 80.6% (25/31), 84.6% (77/91) and 57.7% (15/26) in type 1, type 2 and prog-type groups, respectively (*χ*^2^ = 8.912, *P* = 0.012) (Table 1). However, within each ROP groups, no significant difference was observed between vaginal and C-section rates (all *P* > 0.05). When stratifying by delivery mode, among C-section infants, the proportion of prog-type (12.8%, 15/117) was significantly lower than type 1 (21.4%, 25/117), type 2 (65.8%, 77/117) was significantly higher than that of type 1 (both *P* < 0.05), with no significant difference between type 2 and prog-type. Among vaginally delivered infants, no significant differences in ROP groups distribution were found (all *P* > 0.05) (Table 2).

Median 5-min Apgar score was 8(8, 9) for type 1 ROP, 8(8, 9) for type 2 ROP, 8(7, 8) for prog-type (*F* = 8.403, *P* = 0.015). Bonferroni-adjusted pairwise comparisons showed a significant difference between prog-type and type 2 (adjusted *P* = 0.012), while no significant differences were found between the other groups (Table 1).

Median HH/MNCO duration was 8(3, 17) days for type 1 ROP, 19(5, 31) days for type 2 ROP, and 15(8, 34) days for prog-type (*P* = 0.045), but Bonferroni-adjusted pairwise comparisons showed no significant differences between any groups. Median NIV duration was 5(0, 15) days for type 1 ROP group, 5(0, 14) days for type 2 ROP group, and 18(8, 33) days for prog-type group (*P* = 0.002). Bonferroni-adjusted pairwise comparisons revealed that prog-type had significantly longer NIV duration than both type 1 (adjusted *P* = 0.007) and type 2 ROP (adjusted *P* = 0.002), whereas no significant difference was observed between type 1 and type 2 ROP (Table 1).

The BPD-positive rates were 12.9% (4/31) in the type 1 ROP group, 23.1% (21/91) in the type 2 ROP group, and 50.0% (13/26) in the prog-type group (Table 1). Bonferroni-adjusted column proportion comparisons revealed that the prog-type group had a significantly higher BPD-positive rate than both the type 1 and type 2 groups, whereas no significant difference was observed between the type 1 and type 2 groups (both *P* = 0.004), while no significant difference was observed between type 1 and type 2 (*P* > 0.05) (Table 1). When stratifying by BPD status, among BPD-positive infants, the proportion of trans-type (34.2%, 13/38) was significantly higher than that of type 1 (10.5%, 4/38) and significantly lower than that of type 2 (55.3%, 21/38) (both *P* < 0.05), with no significant difference between type 1 and type 2. Among BPD-negative infants, no significant differences in ROP groups distribution were observed (all *P* > 0.05) (Table 2).

### 3.2 Multinomial logistic regression analysis

Variables showing statistical significance in univariate analysis, including GA, BW, mode of delivery, 5-min Apgar score, HH/MNCO and NIV duration, and presence of BPD, were entered into a multinomial logistic regression model. The analysis was performed with ROP type as the dependent variable, using type 2 ROP as the reference category. The variance inflation factor(VIF) values of all independent variables were < 5, indicating no significant multicollinearity, thus excluding its interference with the regression results (Table 3). Stepwise selection was performed, and the final model showed that GA and HH/MNCO duration were the only variables included in the regression equation. Compared with type 2 ROP, GA (*OR* = 0.592, *95% CI* = 0.456–0.768, *P* < 0.001) and HH/MNCO duration (*OR* = 0.960, *95% CI* = 0.925–0.996, *P* = 0.029) were associated with lower odds of prog-type (*P* < 0.05), HH/MNCO duration use (*OR* = 0.954, *95% CI* =0.918–0.992, *P* = 0.016) was associated with lower odds of type 1 ROP, while GA (*OR* = 0.842, *95% CI* = 0.673–1.051, *P* = 0.129) showed no significant association (*P* > 0.05) (Table 4).

**Table 3:** Collinearity analysis.

Variable	VIF	Tolerance
GA	4.041	0.247
BW	3.245	0.308
mode of delivery	1.202	0.832
5-min Apgar score	1.212	0.825
HH/MNCO	1.657	0.604
NIV	2.136	0.468
BPD	1.402	0.713

**Table 4:** Multinomial logistic regression analysis.

	*β-value*	*S.E.*	*Wald*	*OR*	*95% CI*		*P*
					LB	UB	
Prog-type							
GA	−0.524	0.133	15.507	0.592	0.456	0.768	< 0.001
HH/MNCO	−0.041	0.019	4.775	0.960	0.925	0.996	0.029
Intercept	15.063	4.129	13.308	-	-	-	< 0.001
Type 1 ROP							
GA	−0.173	0.114	2.305	0.842	0.673	1.051	0.129
HH/MNCO	−0.047	0.020	5.533	0.954	0.918	0.992	0.019
Intercept	4.949	3.663	1.826	-	-	-	0.177

Type 2 ROP as the reference category. Model fitness: likelihood ratio *χ*^2^ = 24.753, df = 4, *P* < 0.001, Nagelkerke coefficient of determination (R^2^) = 0.182.

## 4 Discussion

ROP is a multifactorial, early-onset proliferative retinal vascular disease whose development involves complex interactions among numerous risk factors. In this nearly 11-year retrospective study, we analyzed 27 variables retrieved from the medical records of 148 infants with ROP, who were divide into three groups: type1, type 2, and prog-type. Univariate analysis revealed significant differences among three ROP groups in terms of GA, BW, mode of delivery, 5-min Apgar score, HH/MNCO or NIV duration, and presence of BPD. A multinomial logistic regression model was established, which was statistically significant, after assessing collinearity using VIF and tolerance, the final model showed that only GA and HH/MNCO duration were independently associated with severe ROP. Specifically, GA was correlated with prog-type group, while the HH/MNCO duration was associated with both type 1 ROP and prog-type.

Previous studies have demonstrated that low GA is a critical risk factor for ROP (7). In our study, we further compared GA across different ROP groups and stratified subgroups. The distribution of the prog-type was balanced among infants with GA < 27 weeks, 27–32 weeks, and ≥ 33 weeks, with no significant intergroup differences. However, distinct groups characteristics were observed across GA subgroups. In moderately preterm infants with GA (27–32 weeks), primary type 1 ROP was significantly higher than that of prog-type. In contrast, infants with GA ≥ 33 weeks were predominately affected by type 2 ROP, while the proportion of prog-type cases was the lowest. These findings indicate that type 2 ROP tends to occur in more mature preterm infants, whereas prog-type are not uniquely concentrated in the most immature neonates. Nonetheless, prog-type still presented lower average GA and BW compared with type 2 ROP. Multinomial regression analysis further supports that GA remained independently associated with prog-type. An increase in GA was correlated with a reduced the risk of progression from type 2 to type 1 ROP, suggesting that higher systemic and retinal maturity may enhance resistance to ROP deterioration.

Previous research has suggested that a higher incidence of ROP was observed in infants born through vaginal delivery compared with C-section (14). Extending from this observation, we found no significant differences within any groups. Among C-section infants, type 2 ROP was most common, followed by type 1 and prog-type; among vaginally delivered infants, the three groups were similarly distributed. Additionally, prior studies identified low 5-min Apgar score were significant risk factors for the manifested ROP to progress to stages requiring treatment (15). In our study, the prog-type group had a significantly lower median 5-min Apgar score than the type 2 group. However, this variable was not retained in the final multivariate model. Likewise, delivery mode did not enter the multinomial regression equation. These findings suggest that the univariate associations are likely confounded, and neither delivery mode nor 5-min Apgar score emerged as independent risk factors in our study.

Oxygen supplementation is widely recognized as a risk factor for ROP (16). In this study, different modes of oxygen therapy were compared. Univariate analysis showed that infants with prog-type and type 2 ROP had longer exposure to HH/MNCO than those with type 1 ROP. In addition, infants with prog-type had longer NIV duration than those with type 2 or type 1 ROP. After adjusting for confounding factors, HH/MNCO was retained in the final regression model and showed a significant negative correlation with both prog-type and type 1 ROP. Clinically, infants receiving HH/MNCO support already represent those with relatively stable systemic conditions. Nevertheless, whether preferential application of HH/MNCO therapy can provide additional benefits under equivalent clinical indications remains to be confirmed by prospective studies. HH/MNCO delivers much lower positive airway pressure than NIV and IMV, and is therefore considered a low-level respiratory support modality in neonatal care (17). Previous studies have suggested that NIV may increase the risk of ROP by exacerbating fluctuations in oxygen exposure, whereas HH/MNCO showed no independent association (18). The negative correlation between HH/MNCO and severe ROP observed in the present study is consistent with this reasoning, as infants with severe ROP tend to require more intensive oxygen support, resulting in a shorter duration of low-level oxygen therapy. However, in this study, the duration of oxygen therapy was calculated based on the diagnostic window of ROP. This difference in observational windows inherently introduces bias in the comparison of cumulative oxygen therapy duration among the groups. Although partial adjustment was achieved by controlling for GA and other variables in the multivariate regression, the systematic effect caused by different recording endpoints could not be fully eliminated.

Notably, 30 ROP cases were diagnosed among preterm infants with GA ≥ 32 weeks, including 8 cases of type 1 ROP. This finding suggests that appropriately expanding the current ROP screening criteria may be considered to reduce missed diagnoses. Additionally, the overall incidence of ROP in our cohort was significantly lower than reported in the literature (1), which may be attributable to Hainan's unique geography and climate. Further investigation is needed to clarify the correlation between environmental factors and ROP incidence.

## 5 Limitations

To date, very few studies have investigated the conditions under which type 2 ROP is more likely to progress to type 1 ROP. As a retrospective study, this research has certain inherent limitations. Firstly, the study variables were confined to those available from medical records, and these unrecorded data may serve as potential confounders. For instance, although postnatal weight gain velocity serves as an important predictor of ROP, 30 of 148 infants with ROP had missing 6-week postnatal weight gain data, which prevented this factor from being included in the analysis. In addition, detailed data on oxygen saturation targets and oxygen fluctuations — potential confounders related to ROP progression — were not fully captured in the retrospective database. Secondly, the small sample size of ROP cases in this study led to reduced statistical precision. Furthermore, the regression model has not been externally validated. Therefore, the findings of this study need to be validated in future multicenter, large-sample, prospective studies. Accordingly, our results should be viewed as preliminary hypothesis-generating findings rather than definietive conclusions.

## 6 Conclusions

Traditionally, clinical attention has focused on type 1 and type 2 ROP as defined by the ETROP classification: type 1 requires immediate treatment, whereas type 2 necessitates close follow-up observation, as a subset of cases may progress to type 1 disease. Identifying the factors the drive such progression during follow-up is therefore an important clinical question. In our 11-year retrospective study, we specifically focused on the prog-type group and analyzed 27 clinical variables. Compared with the type 1 and type 2 ROP groups, the prog-type group had the lowest GA, the lowest BW, the lowest rate of C-section, the longest NIV duration, a high rate of concurrent BPD. In the final regression model, with type 2 ROP as the reference category, only GA and HH/MNCO duration were retained. Both variables were negatively associated with prog-type and type 1 ROP.

## Data Availability

The original contributions presented in the study are included in the article/supplementary material, further inquiries can be directed to the corresponding authors.
